# Confidence intervals: what are they to us, medical doctors?

**DOI:** 10.3325/cmj.2019.60.375

**Published:** 2019-08

**Authors:** Vladimir Trkulja, Pero Hrabač

**Affiliations:** 1 Department of Pharmacology, Zagreb University School of Medicine, Zagreb, Croatia *vtrkulja@mef.hr*; 2 Department of Medical Statistics, Epidemiology, and Medical Informatics, “Andrija Štampar” School of Public Health, University of Zagreb School of Medicine, Zagreb, Croatia

In 2000, a BMJ-edition book (1) straightforwardly pointed-out the importance of providing effect measures with confidence intervals (CI) when reporting the results of clinical/epidemiological research, and not only the results of statistical tests. However, medical doctors commonly seem to be more aware of formal statistical testing and more fascinated with statistical significance than they are aware of the practical meaning of the location and size (and other properties) of the effect measure. The caveats of scientific reasoning based solely on the results of statistical tests and “*the concept of ‘statistical significance’, typically assessed with an index called the P value*” (2) have been thoroughly addressed. Here, we would only emphasize: the effect estimate (together with its CI) provides information that is not conveyed by the simple fact of it being “statistically significant” (*P* < 0.05) or not (*P* > 0.05) (whatever this, in its essence, would mean) (1) – imagine the effects of two preventive interventions, both associated with “*P*<0.05” (“statistically significant”), but one estimated at 5% relative risk reduction (95% CI 1.0 to 9.0) and the other estimated at 30% reduction (95% CI 26.0 to 34.0). This shifts the focus away from testing and onto the questions about practical relevance of the observed effects. This becomes a matter of methodological (how the effects were observed) and medical expertise, not just statistical. However, the process requires that some concepts, including CIs, are adequately perceived, since they could be quite confusing for non-statisticians like medical doctors.

Research tends to identify generally applicable (generalizable) principles about relationships between factors that affect occurrence, natural history/diseases outcomes, or may help distinguish between health and disease, that would support claims like “this lifestyle intervention reduces the risk of type 2 diabetes by 30%”or “if this test is positive, it is 99% probable that the patient is indeed sick.” Such claims pertain to all potentially or actually diseased people at any time (population-wise claims). The inherent obstacle in this process is the fact that one cannot explore these relationships by encompassing the entire population since population is not a fixed “body of people” – every day some members of the population of, eg, patients with chronic heart failure (CHF) die, while some are newly diagnosed. Therefore, one explores relationships between factors within samples from the population (even studies designated as population-based or nationwide are performed in samples) and then projects back to the population, ie, one estimates the population.

In 1937, Polish mathematician and statistician Jerzy Neyman introduced the concept of CIs (3) and defined the problem of estimation in the introductory sentences (quote):

(ia) The statistician is concerned with a population, π, which for some reason or other cannot be studied exhaustively. It is only possible to draw a sample from this population which may be studied in detail and used to form an opinion as to the values of certain constants describing the population π….

… the problem … is the problem of estimation. This problem consists in determining what arithmetical operations should be performed on the observational data in order to obtain a result, to be called an estimate, which presumably does not differ very much form the true value of the numerical character…

(ii) The theoretical aspect of the problem of statistical estimation consists primarily in putting in a precise form certain vague notions mentioned in (i)… connected with the sentence describing the meaning of the word estimate. What exactly is meant by the statement that the value of the estimate “presumably” should not differ very much from the estimated number? The only established branch of mathematics dealing with conceptions bearing on the word “presumably” is the calculus of probability.”

Deriving from the calculus of probability, he demonstrated that it was impossible to assume that the estimate (as a single value) would be “*exactly equal to*” (quote) the population parameter (object of estimation), but rather, that the process needed to result in the estimation of limits “*between which the true value* [of the population parameter] *presumably falls*” (quote) (3). He named the interval defined by these limits – CI (3).

There are two conceptual (or philosophical) views on estimation and probability, Bayesian and frequentist (the former being older and named after the 18th century mathematician Thomas Bayes), with some specifics regarding their respective computational methods. Both have wide applications in different scientific disciplines, each with its proponents who could commonly be defined also as opponents of the other (although the two philosophies are far from opposing). The concept of CIs and the closely related concept of statistical hypothesis testing [also greatly contributed to by Neyman (4)] together with all the subsequent developments in the field are core frequentist concepts (the term frequentist comes from the view that probabilities of events or numerical values are defined by their relative frequency of occurrence in an infinite number of observations, ie, their occurrence in a long-run). Before we try to outline the main points about the two estimation philosophies, we point-out: (i) parameter is a population value that we want to estimate (“the true effect” or “the true value”); (ii) effect or statistic refers to any quantity that we determine in a sample in an attempt to estimate the population value: ie, mean or median of some continuous variable (eg, blood pressure), proportion of people with some characteristic (eg, cured), correlation or a regression coefficient indicating an association between variables (eg, between blood glucose and cholesterol levels), difference in means or medians between groups of subjects, difference in proportions or ratio measures (eg, relative risk, odds ratio, hazard ratio, incidence rate ratio) etc; (iii) population is defined by sets of characteristics, with no time constraints. For example, population of people with CHF refers to both present and future, and we know that there are at least two (sub)populations of CHF patients (5) – those with reduced left ventricular ejection fraction (LVEF) and those with preserved LVEF. Based on their characteristics, one of which is the fact that some interventions convey a considerable survival benefit in the former, but not in the latter, these are two different populations (5).

The main points of the Bayesian approach (6) are (i) parameter is a random variable, hence it needs to be estimated in a form of a probability distribution. This probability, largely but not solely [see (ii)] defined by data observed in the sample, is called posterior probability or posterior probability distribution. The calculation of the posterior distribution is the final result of the Bayesian estimation process. One can define a certain appropriate point of this distribution that is located at the highest density of the probability of the “true effect”, eg, mean or median, ie, the point-estimate, and an interval that contains the “true value” with a certain level of probability. This interval is called the credible interval (CrI), eg, 95% CrI tells us that the true population value is contained between its lower and upper limits with 95% probability. Different types of CrIs (95%) could be constructed, eg, “equal-tail” intervals, where 2.5% probability of the location of the true effect is below the lower interval limit and 2.5% is above the upper limit; or the highest posterior density interval (HPD) – it may not have equal tails, but it is the shortest interval encompassing 95% of the probability and any point contained within the interval “bears” a higher probability of the location of the true effect than any point outside the interval; (ii) posterior probability is determined (calculated) based on three key elements. The first one is the prior probability or prior probability distribution or simply – the prior. It reflects our previous knowledge, or a belief or a hypothesis (H) about the true effect – what we think that the “true value is” before we have seen the data (ie, before we have done the sample-based observation). The second one is the information collected by observing the sample (the data, D). The third element is the likelihood. Probability and likelihood are commonly used synonymously. Here, likelihood means probability (P) of observing exactly what we have observed in the sample, if our prior (our “pre-data” hypothesis, H) were true [likelihood = *P*(D|H)]. The computational part uses these three key elements to derive the posterior distribution – which, in a way, illustrates how our initial hypothesis was modified by the data and is influenced both by the prior and the observation in the sample. Calculation of the positive and negative predictive values (PPV, NPV, respectively) of a diagnostic test is a clear application of the Bayesian method: both PPV and NPV are posterior probabilities, that is, post-test probabilities of a disease if a test is positive (PPV) or of no disease if the test is negative (NPV) – for any combination of sensitivity and specificity (observed in the sample), they vary depending on the prevalence of a disease in the population (ie, the pre-test or prior probability of the disease/no disease). This is the essence. A simple (hypothetical) example: a randomized controlled trial aims to assess whether a treatment T conveys a survival benefit defined as a difference in 1-year mortality vs placebo (control, C) in patients with advanced stages of CHF with reduced LVEF who are already on their standard therapy. Considering the clinical setting and a potential meaningful effect, it is estimated that 2000 patients need to be included and randomized to T and to C in a 1:1 ratio, with randomization stratified by age and clinical severity stage (since both affect life expectancy). At the end, 1-year crude cumulative mortality is 20% with C and 16% with T. The T-C difference needs to be estimated with adjustment for age and baseline disease severity, ie, in a generalized linear model that models ln(risk) of death with T and with C to determine the difference [ln(riskT)-ln(riskC)] (exponentiation of the difference yields relative risk, RR). Bayesian analysis: a) two options may be considered, (i) one with no relevant pre-study input that would result in a meaningful (the so-called informed) prior, hence a so-called non-informative prior is used, and (ii) another one using an informed prior based on a pilot study indicating around 25% relative risk reduction and suggesting 95% probability of the effect being within the range between a 62.5% lower and a 50% higher relative risk; b) based on our input (prior and the data, ie, treatment, outcome, age, and disease severity), the computational algorithm uses complex simulation processes to generate and to randomly sample from a large number of simulated distributions to estimate the posterior distribution of ln(riskT)-ln(riskC): exponentiation of particular points of the distribution retrieves RR (median), equal-tail CrI (2.5 and 97.5 percentile), or HPD CrI. Based on the results (Table 1), we conclude that the risk of 1-year mortality with T is relatively 20% lower than with C and that it is 95% probable that the effect is in the range between 34.4% lower and 4.8% lower. This is our claim about T. Note: SAS 9.4 for Windows (SAS Inc., Cary, NC) proc genmod (log link, binomial distribution) was used with (i) the built-in option for Bayesian analysis with Jeffreys prior; or (ii) a normal prior with mean -0.288 and variance 0.125 for treatment (0, 1e6 for other effects). Visual inspection of the trace plots indicated good Markov chain convergence. The use of informative or non-informative priors in data analysis is a question of debate. In general, for data like the present hypothetical trial, non-informative priors are preferred. Similarly, the preference is toward HPD vs equal-tail CrIs (*https://www.berryconsultants.com/use-bayesian-trial/*, accessed June 15, 2019).

**Table 1:** Results of the Bayesian analysis of the hypothetical randomized trial comparing treatment (T) to control (C) regarding 1-y mortality in patients with advanced chronic heart failure. Results are relative risks (RR) with highest posterior density (HPD) or equal-tail (2.5 to 97.5 percentile) 95% credible intervals (CrI) for two scenarios: one using a non-informative prior (Jeffreys) and another one using a normal (mean -0.288, variance 0.125) prior probability distribution for treatment

	RR	95% CrI (HPD)	95% CrI (2.5-97.5)
Non-informative prior	0.800	0.656-0.952	0.664-0.964
Informative prior	0.829	0.682-0.981	0.692-0.993

**Figure 1:**
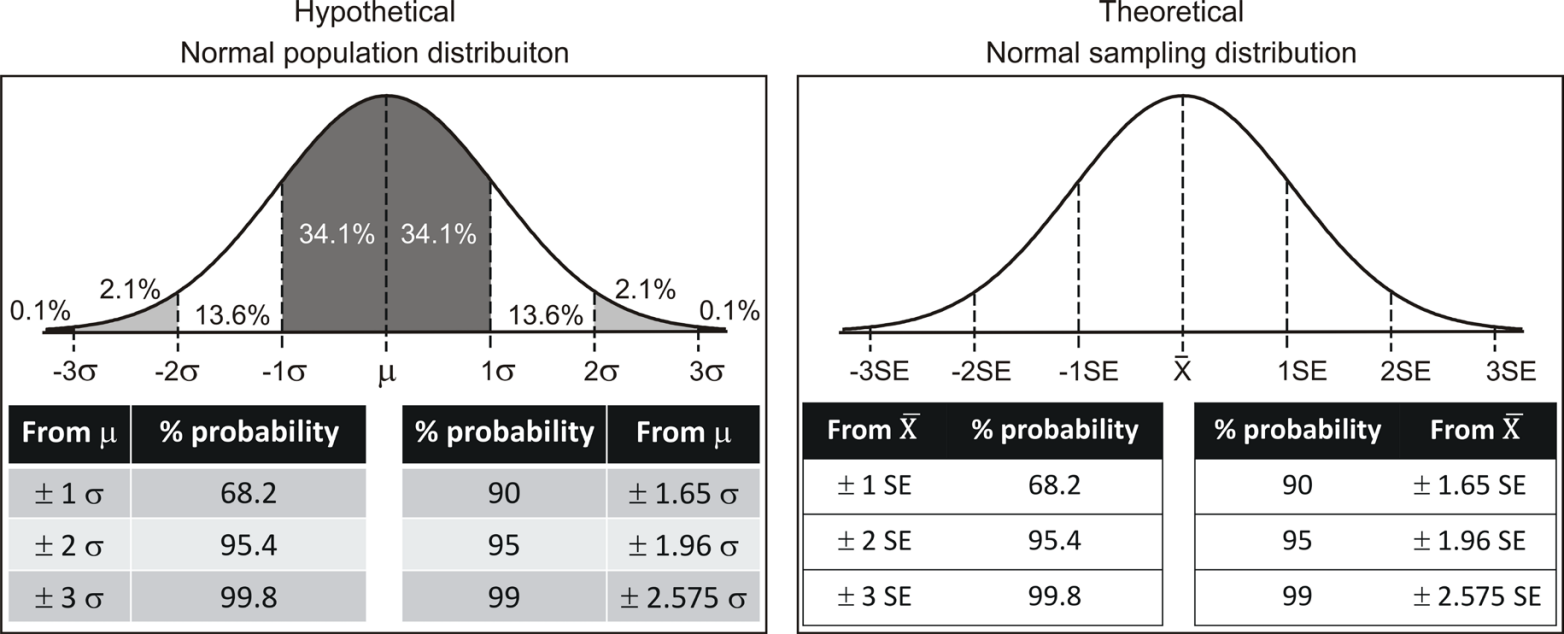
Graphical representation of normal distribution, ie, “the bell-shaped curve” (probability density function). The curve on the left is designated as “population distribution,” a hypothetical one – since no research ever encompasses the entire population. Two parameters that define it are its center, ie, population mean (m; to be distinguished from a mean value of some continuous variable determined in a sample from the population 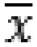) and a measure of dispersion of probability around the mean, ie, population standard deviation (σ; to be distinguished from a standard deviation of some continuous variable determined in a sample from the population – SD). The table below indicates the percentage of probability contained in intervals defined as deflections from the mean expressed as multiples of σ added to or subtracted from the mean. The curve on the right is exactly the same, but is designated as a theoretical one (may be derived by mathematical simulations based on the sample data) and is named sampling distribution as it represents the distribution of statistics (estimates of m) observed in an infinite number of independent random samples from the population. The difference vs population distribution is pointed-out by the used symbols: 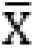 for the mean and SE (standard error) for the standard deviation. The two parameters of the sampling distribution are estimates of the respective parameters of the population distribution and it is assumed (since referring to the distribution of estimates from an infinite number of independent random samples from the population) that 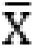
=m and SE = σ. See text for further explanations.

The frequentist approach. The originating work (3) intended to define an estimation theory that would be independent from the Bayesian solution (ie, use of priors), which was dominant at the time (4). Main points (1,3,7,8) are (i) the parameter is a fixed but unknown value. In the CHF example – there is one constant “true population value” of the RR for T/C regarding 1-year mortality (true *RR*). No prior probability is assigned to it and it is not estimated in a form of a probability distribution. It does not mean that we do not have “a clue” about what its value could be –this is just how the concept works. If we could encompass the entire population, then RR in the trial would equal the true RR; (ii) but we cannot, and we base the estimation on a sample. The sample is a random sample from the population; (iii) hence the estimate (the statistic, RR) determined in the sample is a random variable (unlike the parameter). If we were to draw a large number of independent random samples of the same size from the same population (studies; where not a single subject is included in more than one of the samples), and repeat the estimation process in exactly the same way, the estimates obtained in each of them would somewhat differ, simply by chance. Hence, the estimate (the statistic) is a random variable and has a certain probability distribution; (iv) probability distribution of the statistic is called sampling distribution, and the by-chance variation of the estimates that constitute the sampling distribution is called sampling variation. Several points might be confusing. For example, if the population is not a “fixed body of people,” how can the sample be random when we cannot sample from the future? Even if one was to limit the population to the specific moment in time – what would random mean? In epidemiology, particularly, there is a whole branch dealing with the (random) sampling procedures. For example, if one is interested in the nationwide status of some disease or an opinion on some political issue in 2019, there are methods that enable generation of the sample of the population 2019 that is random and, hence, representative of thus defined population. In clinical studies, however, we never have a random sample of patients (not even of those “potentially available at the time”). But, we behave as if we did and there are things that can and need to be done to ascertain that the sample (of subjects) included in a study fairly well represents the population. Next, in reality, for some “true values” we only have one estimate: eg, one large clinical trial for a new treatment in CHF patients with a statistic determined only once (5). How can one numerical value be “distributed”? Sampling distribution is an abstract construct and could be determined for any statistic by mathematical procedures. It just happens so that sampling distribution of practically any statistic is a normal or near-normal distribution: if the population distribution is normal – sampling distribution is normal regardless of the number of observations; if the population distribution is not normal, sampling distribution becomes near-normal already with ∼ 20 observations (says the Central Limit Theorem). We use a simple example that illustrates the general principle. Let us define our population of interest as a finite one, say “10-year old boys in the country in 2019,” and let us say that the characteristic of interest is their height. Each member of the population has its own value, ie, height (in cm). Distribution of their individual values is the population distribution. It is a normal distribution and one of the parameters that define it is its “center” or the mean – population mean or mean of the population distribution (m). This is the fixed value (“true value”) that we want to estimate. To do so, we take a random sample from the population and determine the sample mean (the statistic). However, the “link” between the sample and the population is not a direct one – sampling distribution of the sample statistic is an “in-between” step that connects them: the sample mean (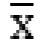) is a realization of the random variable height (conceived as a range of 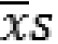 calculated in a large number of independent random samples of the same size taken from the same population), ie, of the mean of the sampling distribution of 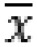. The mean of the sampling distribution (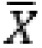) is, hence, a statistic of a statistic (mean of the distribution of means from many samples). The “link” between the sample statistic 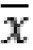 and the population parameter m is in the fact that 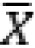 (statistic of the sample statistic) = m. So, using a sample, we calculate 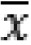 which is the point-estimate of 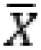
=m. It might not be clear why would one need this “in-between” step. Note: even if finite, the entire population is never encompassed; population probability distribution is a hypothetical one; its mean, our “target” is fixed, but we try to estimate it using random samples, hence estimates (sample-based statistic) vary (sampling variation); while point-estimation is rather straightforward, the fact that estimates are random variables (hence have a probability distribution) is the key point – one needs a framework for mathematical operations that enable one to account for this fact in defining the interval estimate of the “true value”; (v) another term that is a part of this theoretical concept that needs to be mentioned is sampling error (9). Assuming that we indeed took an “ideally” random sample (hence, representative for the population, ie, “population in a nutshell”), the sample-based statistic would deflect from the “true value” purely by chance, ie, due to the sampling variation. Namely, the fact that statistics from different samples differ (by chance) from each other means also that each of them inherently differs from the “true value.” Sampling error refers to the distance of the sample statistic from the “true value.” It is only natural that one would like to know how much the observed sample value deflects from the “true value,” ie, what its (sampling) error is. This question reflects the uncertainty about the relationship between the point-estimate and the “true value.” Sampling error is a theoretical construct since we would need to know the “true value” in order to be able to directly determine how much the estimate differs from it. Yet, we can assign a quantity to the sampling error by using the sample and the concept of sampling distribution. But, note – this entire concept is based on assumptions of an “ideally” random sample and a deflection that has no other sources but pure chance, and on a theoretical distribution of an “infinite number of such samples.” To continue with the simple example of the sample mean (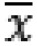), Figure 1 depicts the relationship between a hypothetical population distribution and the sampling distribution*:* a) population mean, m, is the “true value”; b) another parameter that defines normal distribution is a measure of dispersion of probability “around” the mean of the distribution – population standard deviation (σ); c) mean of the sampling distribution (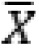) is an estimate of m (assumes 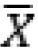
=m); d) standard deviation of the sampling distribution – a measure of dispersion in the sampling distribution that is an estimate of σ is called – standard error (SE). Standard error can be quantified using data in the sample, ie, it is another sample-based statistic and its numerical value illustrates the sampling error. We already established that 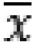 → 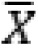. Regarding SE, there are different methods for calculation for different statistic/effect measures (1), but the generally followed logic is as follows: use the measure of spread in the sample → estimate the measure of spread of the sampling distribution. The example of the SE of a sample mean (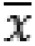) [SE *= SD*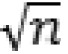, where SD is sample standard deviation, ie, measure of spread in the sample, and *n* is the number of subjects in the sample] illustrates two general principles: (a) for any given number of subjects in the sample, larger the variability of the values in the sample – larger the SE; (b) for any given variability observed in the sample, larger the sample – lower the numerical value of SE (1). In other words, (b) tells that the concept implies that with a larger sample the error, ie, “deflection of the point-estimate from the true value” would be smaller. This sounds intuitive since, as we said, if the sample could encompass the entire population, then the estimate would be = true value, ie, there would be no error; (vi) sample point-estimate and its SE are used to construct the CIs around it. Different methods are used for calculation of the CIs around different statistic/effect measures (1). The example of the sample mean and CIs around it outlines the general principle that the distances of the lower and the upper limits from the point-estimate are in this or that way defined as multiples of the standard error. From Figure 1, it is quite obvious that in the case of the sample mean, the 95% CIs are 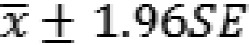. It is also obvious that for 90% CIs or for 99%CIs, different multiples of SE would be used, and for the given SE the intervals would be narrower (90% CI) or wider (99% CI) than the 95% CI. Two further points (cannot be perceived from Figure 1): a) in cases in which the assumption of a near-normal sampling distribution (eg, Student’s *t*-distribution) is more appropriate than the assumption of a normal distribution, similar but different multiples of SE would be used to construct CIs, and these would depend also on the number of subjects in the sample (study) (1); b) for some statistics, CIs around the point-estimate are symmetrical, for some they are not (1); (vi) to get back to the T vs C trial in CHF patients: a) there are no priors, ie, estimation of the population value is based solely on what is observed in the sample; b) we implement appropriate calculation method, ie, “frequentist” analysis of a generalized linear model to obtain the measure of the effect of T (adjusted for age and disease severity). Again, “everything is done” with ln(riskT) and ln(riskC) and their difference: results are summarized in Table 2. Based on the results, we conclude that the risk of 1-year mortality with T is relatively 20% lower, with 95% CI from 33.7% lower to 3.4% lower. The estimate is closely similar to the Bayesian point-estimate and CrI. In this and many cases, frequentist CIs and Bayesian CrIs could be identical or closely similar, but in many – they would not (particularly when the so-called informative priors are used) (6,8). Note: SAS for Windows 9.4 (SAS Inc., Cary, NC), proc genmod with log link and Possion distribution, with a robust (“sandwich”) variance estimator.

**Table 2:** Results of the frequentist analysis of a hypothetical randomized trial comparing treatment (T) to control (C) regarding 1-year mortality in patients with advanced chronic heart failure. Calculations are based on ln(risk): mean difference ln(riskT) – ln(riskC) = -0.2232; standard error = 0.0962; since the sampling distribution of the ln(relative risk) is normal, 95% confidence interval (CI) for the difference = -0.2232 ± 1.96 × 0.0962, ie, -0.4116 to -0.0347. Relative risk (RR) and its 95% CI are obtained by exponentiation of these values

	RR	95% CI
T vs C	0.800	0.663-0.966

With both the Bayesian and the frequentist approach we obtained a point-estimate – and this is our best estimate (given the data) of the “true value.” There is, however, one obvious difference: for CrI we stated it was 95% probable that it contained the “true value” (based on the whole process we undertook), while here, we make NO probabilistic statement related to the calculated 95% CI. We are tempted to do so, just as for the Bayesian CrI: such a statement would be a “natural answer” to a “natural question” (6) – we wanted an answer to the question “does T convey any survival benefit?”; for this purpose, we did the trial; when the trial is done and the estimate generated, the natural question is – does the interval contain the true value, or – how probable it is that it does? Next, our (medical) practice is in its essence largely Bayesian (10). For example, by taking medical history and by examining a patient, we gradually form an opinion about the probability of a certain diagnosis (vs other possibilities) that drives our decisions about subsequent diagnostic tests (in essence – this is the Bayesian prior probability) and affects our opinion about the probability of the respective diagnosis after seeing the test results (the posterior probability): eg, a slightly elevated value of some laboratory parameter might diminish the probability of a diagnosis when the preceding knowledge (medical history, physical examination) indicated a low probability of a diagnosis (low prior probability) or be considered as confirmatory for the diagnosis when previous knowledge strongly suggested the diagnosis (high prior probability). So, we are continuously dealing with probabilities, and thus are, likely, inherently prone to perceive the CI around the point-estimate as a probability statement. There has been a lot of discussion about what the frequentist CIs are and what they are not (6,8,11,12), commonly involving the debate about CrIs vs CIs, ie, Bayesian vs frequentist views (and which is better for science). But, even if one is to stay within the frequentist framework – many different misinterpretations of CIs have been generated (12). One of the most common ones is exactly the one of assigning a probabilistic statement to CIs in the way it is done with Bayesian CrIs (12) – and appears to be equally common among statisticians and non-statistician scientists and students (11,13). However, it is not correct and was not originally claimed (3,14,15). We need to go back to the essence of the concepts: (i) with Bayesian approach, the parameter is a random variable, hence needs to be estimated by a probability distribution. The point-estimate and the limits of CrI are fixed – they are points of a generated posterior distribution – hence, “CrIs make a direct probability statement about themselves”, ie, that it is 95% probable (for 95% CrIs) that they, based on the entire completed process, contain the true value; (ii) with frequentist approach, parameter is a fixed point, it does not have a probability distribution. However, the point-estimate and CIs around it are random variables – they have a theoretical probability distribution (sampling distribution) based on which, for the given study, they are generated. Once calculated, CIs either cover or do not cover the true value – we cannot make any probability statements about it based on the sole fact that we calculated them. Since the sampling distribution based on which they are derived is composed of “many samples, with many respective estimates and CIs” that differ one from the other, one also cannot say that 95% of the point-estimates (from the very same sampling distribution) would fall within the one particular 95% CI that we have determined (12). What we can say and what is in line with the frequentist philosophy that defines probability as relative occurrence of events/values over a large number of repeated observations is – if the underlying model is correct and the only source of variability is chance (sampling variation) alone (ie, there is no systematic error in the process), then if one is to repeat the entire (valid) process in an unlimited number of independent random samples of the same size and from the same population – at least 95% of thus generated CIs would cover the true value (or 90% or 99% – if these are the CIs that are of interest) (8,12). So, a specific CI also “conveys” a “probability message,” but not about its own probability of containing the true value, but about coverage probability – ie, probability that the unobserved (“unlimited” members of the respective sampling distribution) intervals generated by the same valid procedure do cover the true value 95% percent of the time. In other words, the “95% probability*”* refers to the procedure, not to the one specific calculated CI. So, what does this mean for us at the very moment of looking at the obtained estimate? What are CIs to us? Why would we care about possible outcomes of unobserved repetitions that anyhow are only hypothetical? How should we view CIs calculated in this particular study? In their “statistical essence,” CIs are indicators of uncertainty about the point-estimate (remember SE as a “measure” of sampling error, ie, distance of the point-estimate from the true value), which is (uncertainty) due to chance alone, but may be problematic because they depend on the correctness of probability models and sampling properties (8). Some authors consider CIs to be useless, mainly due to their close relationship to hypothesis testing and “*P* values,” and suggest they should be abandoned in favor of Bayesian CrIs (11). Regarding the interpretation of (95%) CIs, the 2000 BMJ-edition book (1) states (p.17): “*Put simply, this means that there is 95% chance that the indicated range includes the ‘population value’…,* while another paper (12), co-authored by one of the co-authors of the cited quote, points-out such an interpretation as incorrect (quote: [misinterpretation No. 19] ”*The specific 95% confidence interval presented by a study has a 95% chance of containing the true effect size*. – No!”). The same author, in another source (16), along with a strictly correct definition of CIs states: “*Little is lost by the common but less pure interpretation of the CI as a range of values within which we can be 95% sure the population value lies*.”

Hence, apart from the message that the frequentist CIs and Bayesian CrIs convey different information, and that the information conveyed by the former is not the one we commonly think it is – everything else is rather confusing. We may become even more confused when we consider the following two facts: a) Bayesian methods have had wide applications in biomedicine, particularly in the analysis of large complex data (eg, those produced by genomic and other *omic* analyses) (17), in pharmacokinetic-pharmacodynamic modeling, meta-analysis, their use in clinical trials is growing, particularly in adaptive-design sequential trials, and similar is the situation in epidemiology (18); b) however, frequentist methods have predominated, in eg, clinical and epidemiological research. We will skip the question whether this is “good or bad” [because (i) it is beyond our reach; (ii) the answer might be neither, or one or the other or even a combined approach might be better, depending on the problem addressed; (iii) because in some situations CIs and CrIs not only agree but could be interpreted in the same way (6,8,17,19,20)], and address another one – does it mean that we have been continuously wrong (since relying mainly on frequentist methods) about relationships of interest? Over the decades, a huge number of estimates of the “frequentist type” have been made resulting in decisions (diagnostic, therapeutic, prophylactic) that have greatly improved medical practice. So, the concept seems to work. Whether and how statistical estimation could be improved is beyond our reach. However, in respect to specifically the concept of CIs it seems that, for all practical purposes, medical doctors who need to read and understand published papers on a daily basis could well accept, without “major harm,” the view expressed (1,16), although also dismissed (12), about interpretation of CIs from a single study (regardless of how conceptually and factually incorrect it might be): “*Little is lost by the common but less pure interpretation of the CI as a range of values within which we can be 95% sure the population value lies*” (16). But it needs to be added: the view that it is plausible to be “95% sure” that one specific actually realized CI that belongs to a range of hypothetical CIs, 95% of which (presumably) do cover the true value, includes the “truth” would hold only if “the process,” ie, the way in which data were collected and analyzed was a valid one. By saying this, we need to move away from the strictly statistical (conceptual and computational) views about “estimation of reality,” regardless of how essentially important they are, toward the equally important elements of the process that are inherently easier for us to understand. We will assume that in any individual study, statisticians have done their work correctly, that they have chosen the adequate approach and methodology for data processing, and that corresponding interval estimates (CrIs or CIs) are provided. The point is – while this part is clearly important, no statistical method can mend the flaws that occur in the process of data gathering (be it an experimental or observational study) – if data are invalid, the estimates (whatever their theoretical basis) will be invalid. So, we need valid data in order to get estimates that are “on target.” An inherent consequence of the fact that no one actually knows what the true value is, is that there is no immediate way to check this– eg, years could go by before we realize that an intervention is not as effective or that it is more harmful than initially estimated. Off-target estimates come as a consequence of systematic (bias) and random errors in the process of data gathering and estimation: this is the question of research methodology. Therefore, we need to be familiar with research methodology: types of studies appropriate for respective questions, their reaches and limitations, types of various biases to which such studies are sensitive, and methods of “protection” against them. A point-estimate generated in a study that, by type and design, has a potential to be accurate and could be judged as “well protected from bias” – is highly likely to be close to the “true value,” although we might not necessarily be able to say “how close” and “how likely” (or express this as, eg, a percentage). The interval around it (be it Bayesian or frequentist, 90%, 95% or 99%, or any other) would then be highly likely to cover the “truth.” We would therefore state that CIs (around estimates) provide important information that goes beyond the results of statistical tests and *P* values, regardless of their inherent close relationship to statistical testing (and *P* values) and their inherent conceptual limitations/fallacies. They should always be viewed within the entire context – for a study (experimental, observational or meta-analysis), this refers to its general appropriateness for the addressed question, design, and conduct characteristics and, of course, adequacy of the implemented statistical procedure. If these elements, ie, this “methodological package” could be considered valid – then we may consider the resulting CI around the estimate as a reliable indicator about the location and size of the true value, and thus, as a reliable basis to consider the practical relevance of this (true) value, regardless of the results of statistical tests. Although it may sound heretic, under such circumstances, for all practical purposes it becomes of less relevance to us whether the interval is a direct probability/certainty/confidence statement or not. At the end, we build our final “certainty” or “confidence” about size of the true effects based on (i) independent replication of estimates arising from methodologically adequate procedures and (ii) concordant estimates from different types of studies.
